# Large-scale patterns of benthic marine communities in the Brazilian Province

**DOI:** 10.1371/journal.pone.0198452

**Published:** 2018-06-08

**Authors:** Anaide W. Aued, Franz Smith, Juan P. Quimbayo, Davi V. Cândido, Guilherme O. Longo, Carlos E. L. Ferreira, Jon D. Witman, Sergio R. Floeter, Bárbara Segal

**Affiliations:** 1 Programa de Pós-Graduação em Ecologia, Universidade Federal de Santa Catarina, Florianópolis, Santa Catarina, Brazil; 2 Marine Macroecology and Biogeography Lab, Departamento de Ecologia e Zoologia, Universidade Federal de Santa Catarina, Florianópolis, Santa Catarina, Brazil; 3 Laboratório de Ecologia de Ambientes Recifais, Departamento de Ecologia e Zoologia, Universidade Federal de Santa Catarina, Florianópolis, Santa Catarina, Brazil; 4 Department of Ecology and Evolutionary Biology, Brown University, Providence, Rhode Island, United States of America; 5 Marine Ecology Lab, Department of Oceanography and Limnology, Universidade Federal do Rio Grande do Norte, Natal, Brazil; 6 Reef Systems Ecology and Conservation Lab, Universidade Federal Fluminense, Niterói, Rio de Janeiro, Brazil; Department of Agriculture and Water Resources, AUSTRALIA

## Abstract

As marine ecosystems are influenced by global and regional processes, standardized information on community structure has become crucial for assessing broad-scale responses to natural and anthropogenic disturbances. Extensive biogeographic provinces, such as the Brazilian Province in the southwest Atlantic, present numerous theoretical and methodological challenges for understanding community patterns on a macroecological scale. In particular, the Brazilian Province is composed of a complex system of heterogeneous reefs and a few offshore islands, with contrasting histories and geophysical-chemical environments. Despite the large extent of the Brazilian Province (almost 8,000 kilometers), most studies of shallow benthic communities are qualitative surveys and/or have been geographically restricted. We quantified community structure of shallow reef habitats from 0° to 27°S latitude using a standard photographic quadrat technique. Percent cover data indicated that benthic communities of Brazilian reefs were dominated by algal turfs and frondose macroalgae, with low percent cover of reef-building corals. Community composition differed significantly among localities, mostly because of their macroalgal abundance, despite reef type or geographic region, with no evident latitudinal pattern. Benthic diversity was lower in the tropics, contrary to the general latitudinal diversity gradient pattern. Richness peaked at mid-latitudes, between 20°S to 23°S, where it was ~3.5-fold higher than localities with the lowest richness. This study provides the first large-scale description of benthic communities along the southwestern Atlantic, providing a baseline for macroecological comparisons and evaluation of future impacts. Moreover, the new understanding of richness distribution along Brazilian reefs will contribute to conservation planning efforts, such as management strategies and the spatial prioritization for the creation of new marine protected areas.

## Introduction

Understanding how marine biodiversity varies on local and regional scales serves as the foundation for studies in ecology, biogeography, and conservation [1–2]. One of the most pervasive large-scale patterns of biodiversity is the latitudinal diversity gradient, in which the highest richness commonly occurs towards the equator and declines towards higher latitudes [3–7], a pattern that has been described for many groups of organisms in terrestrial and marine environments [4,7]. Despite the existence of a relatively consistent pattern across different groups, the latitudinal diversity gradient is somewhat variable among taxa and regions [2, 6]. For example, marine diversity patterns of fish and invertebrates in the Atlantic differ between eastern and western shelves [8] and between northern and southern hemisphere [9–10]. Despite these examples, we still lack a comprehensive and quantitative description of large-scale patterns of benthic communities in the Atlantic.

The Brazilian Province comprises almost 8,000 kilometers of Brazil’s coastline and the offshore islands of Rocas Atoll, Fernando de Noronha, St. Paul’s Rocks and Trindade [11–13]. This region exhibits a wide range of reefs habitats, ranging from the Amazon River mouth (0° latitude) to the state of Santa Catarina (28°S latitude) [11–12, 14]. The region is bounded by three prominent biogeographic barriers: the Amazon Plume, that divides the marine fauna and flora of Brazil from the Caribbean Province; the Mid-Atlantic Barrier, that isolates the Brazilian Province from Western Africa; and low temperatures from the La Plata River plume that limits the distribution of tropical marine organisms southwards [12]. Despite the large spatial extension and heterogeneity, most studies have been conducted on a few areas and over small geographical scales [14–19]. Previously, latitudinal comparisons of benthic community structure along the Brazilian coast have been based on literature reviews and qualitative work (see [20–28]).

Marine ecosystems in many areas worldwide are declining due to anthropogenic impacts, including climate change [29–30]. Many coral reefs have lost their ability to recover after a disturbance, causing phase shifts in benthic structure, such as dominance by macroalgae [31]. Management and conservation efforts have been focused on mitigating the threats to marine environments. In 2010, the Brazilian Government agreed with the targets of the United Nations Biodiversity Convention to protect marine and coastal biodiversity and to establish 10% of marine environments as no-take by 2020; currently, many important coastal ecosystems in Brazil are not protected. Many factors limit our ability to describe, predict and evaluate changes in these ecosystems in order to inform their protection. For example, one of these limitations involves the lack of quantitative baselines and an incomplete understanding of spatio-temporal variation in benthic community structure for many reef systems [32]. Consequently, large-scale information on reef biodiversity of the Brazilian Province provides a critical first step that is needed to establish conservation targets and to mitigate human impacts on these ecosystems.

The aims of our study were: 1) to provide a quantitative, community-wide description of shallow benthic marine communities in the Brazilian Province, and 2) to quantify the biodiversity patterns of the benthic communities along the latitudinal gradient in the Brazilian Province. This study provides a valuable baseline for benthic communities along the Brazilian Province, allowing comparisons on how benthic communities change over time and contributes to the understanding of patterns of reef benthic communities in the Atlantic.

## Materials and methods

### Study area

The Brazilian Province has an extensive coastline and exhibits a wide range of environments. Even though reefs of the Brazilian Province represent only 5% of Atlantic reefs, rates of endemism are high: ~34% for reef-building corals, 11% for macroalgae, and 35% for sponges [22, 33]. The northeastern and central portions of the Province contain carbonatic and sandstone outcrops (mostly biogenic reefs), while the southeastern-southern part is dominated by siliciclastic bottoms on the shelf and sand beaches interrupted by crystalline rocky shores [34]. The Brazilian coast is influenced by the warmer Brazil Current flowing southwards (ocean temperature above 20°C) and the colder Brazilian Northern Current flowing northwards (temperature below 16°C) [35–37]. The southeastern coast is also influenced by upwelling events, especially in Rio de Janeiro and Santa Catarina states, bringing colder and nutrient-rich waters into shallow water environments. This Province is also characterized by high terrestrial runoff from rivers [38], strong wind and variable shelf width [25]. Four oceanic islands belong to the Brazilian Province, three of which were included in the present study: Rocas Atoll (3°87’S; 33°80’W), Fernando de Noronha (3°86’S; 32°43’W), and Trindade Island (20°51’S; 29°33’W).

### Ethics statement

This study was conducted in accordance with all Brazilian government legislation. This includes authorization to the SISBIOTA-Mar project to assess images of the benthic communities along the Brazilian reefs, under the permits # 06/2012 (Parcel do Manuel Luis; SEMA-MA), # 29953–1 (Rocas Atoll; ICMBio/ MMA—Brazilian Ministry of Environment), # 29687–2 (Fernando de Noronha; ICMBio/ MMA—Brazilian Ministry of Environment), # 32145–1 (Costa dos Corais, ICMBio/ MMA—Brazilian Ministry of Environment), # 22637 (Abrolhos, ICMBio/ MMA—Brazilian Ministry of Environment), # 4416–1 (Trindade Island, ICMBio/ MMA—Brazilian Ministry of Environment), # 37869 (Alcatrazes, ICMBio/ MMA—Brazilian Ministry of Environment), # 21422 (Florianópolis Norte, ICMBio/ MMA—Brazilian Ministry of Environment), and for RN Maracajaú (APA dos Recifes de Corais, IDEMA-RN).

### Benthic sampling

**Fig 1:**
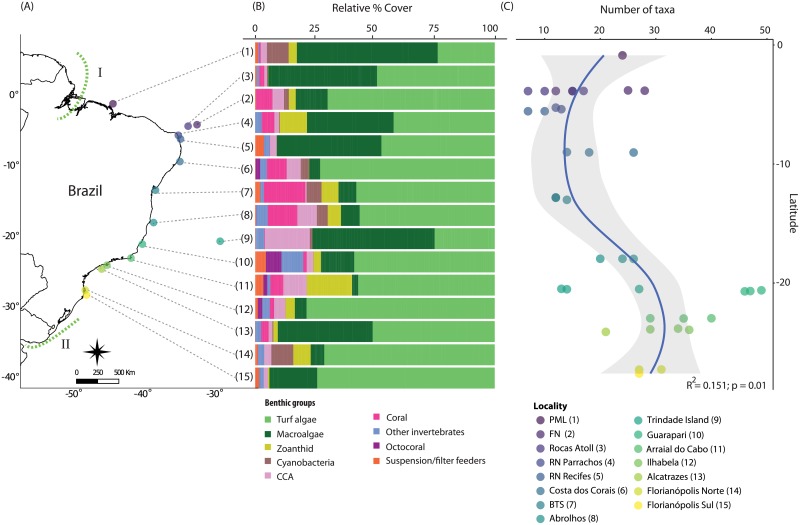
Relative percent cover of benthic groups and richness along the Brazilian Province. (A) Location of study localities in the Brazilian Province, (B) Relative percent cover of benthic groups by localities (non-living organisms excluded), (C) Number of taxa by sites along the Brazilian Province. The blue line represents the second-order polynomial equation. I = Amazon discharges and II = La Plata River plume. PML = Parcel do Manuel Luis, FN = Fernando de Noronha, BTS = Baia de Todos os Santos.

We sampled 40 sites within 15 localities from 0° to 27°S latitude along the tropical and subtropical reefs of the Brazilian Province during the austral summer from 2011 to 2014 (Fig 1A; S1 Table). Seven localities were located on biogenic reefs, and eight were rocky reefs (S1 Table). At each locality, between one and five sites were assessed (but most had at least three sampled sites; S1 Table). At each site, surveys were conducted at two depth strata: 1–7 meters (shallow) and 8–15 meters (deep), unless only one-depth strata was found. We haphazardly selected six to twenty 2m^2^ horizontal surfaces of reef area on each depth strata (S1 Table) and characterized the benthic community using a set of five 25x25 cm photoquadrats [19]. The 2m^2^ areas were at least 2 meters apart from each other, and were treated as independent samples in the analysis. We used the 2m^2^ areas method to sample comparable horizontal surfaces on reefs. Some sites were composed by big boulders where transects would be hard to use and we would have to include vertical surfaces in the sampling. This type of bias associated with the 2m^2^ method is very similar to those observed in traditional transect methods. Between 8 to 30 reef areas were assessed at each site, resulting in a minimum of 40 and maximum of 150 photoquadrats per site representing a total of 3,855 photoquadrats sampled in the entire study (S1 Table).

### Photoquadrat analysis

Images obtained from the photoquadrats were analyzed using photoQuad software [39] by laying fifty random points on each image and identifying the organism underneath. The identification of benthic organisms simply using images can be problematic, with loss of taxonomic resolution [40]. Some groups would require destructive sampling, complex and time consuming techniques to achieve lower taxonomic resolution. Therefore, all organisms were identified to the lowest taxonomic level possible (adapted from [41]). This resulted in taxa identified to different hierarchical taxonomic levels, where 82% of taxa were able to be identified to Family level (S2 Table). We are aware of the potential problems associated to mixing different taxonomic resolutions, however this approach would tend to make our diversity results conservative rather than exaggerated. In other words, it could change the magnitude but not the direction of the observed patterns. We recognize the trade-off between our goal of studying the entire benthic community versus loss of taxonomic precision. Published guides, checklists (e.g. [42–45]), and taxonomic specialists were frequently consulted during the analysis of these images to confirm accurate identification. Protocol is available on the protocols.io: dx.doi.org/10.17504/protocols.io.p2wdqfe. Raw data and the classification scheme are available on the Dryad repository (doi:10.5061/dryad.f5s90).

#### Benthic structure

For benthic community composition, percent cover data (organisms classified at the lowest taxonomic level possible) were transformed by arcsine-square root, to reduce the influence of abundant and rare organisms [46]. We compared benthic community composition among localities by cluster analysis (complete linkage method) by using the function *pvclust* within the package “pvclust” [47] in R software [48]. A cophenetic correlation analysis was used to calculate the reliability of cluster branches. Additionally, we evaluated differences of community composition between depth strata by sites nested within localities by nonmetric multidimensional scaling analysis (NMDS) with Bray-Curtis dissimilarity using the function *metaMDS* within package “vegan” [49]. Statistical differences in community composition were tested between depth strata, reef type (biogenic and rocky reef) and localities (only for sites with both depth strata sampled) with PERMANOVA analysis using the function *adonis* within the package “vegan” [49] in R software [48]. The statistical significance of the PERMANOVA was tested using 999 permutations under a reduced model and type II (conditional) sums of squares [50].

To analyze community structure in terms of the dominant groups of biota we also grouped the percent cover of benthic organisms into nine benthic groups associated with resource use and their capability to respond to different environmental conditions (e.g. light, food, space). These were: crustose coralline algae (CCA), coral, cyanobacteria, macroalgae, octocoral, other invertebrates, suspension/filter feeders, turf algae, and zoanthid, and we showed their latitudinal patterns by localities and sites by depth strata. Algal turfs are a recognized major component of reef environments and can be defined as a complex epilitical algal matrix, which includes detritus/sediment and cryptofauna associated [51–52].

#### Diversity patterns

We used the number of all taxa (observed) from each site to evaluate trends of diversity along the latitudinal gradient of the Brazilian Province, with sites nested within localities. Species richness estimations were calculated for the Chao metric (observed plus undetected taxa) to compare across sites with different levels of sampling intensity [53]. Species accumulation curves were built using the function *poolaccum* and *specpool* within package “vegan” [49] in R software [48]. We used the package “ggplot2” to plot the number of taxa and latitude, using the function *stat_smooth* to identify the patterns [54] and the function *poly* within package “stats” to perform regression analysis [48]. All statistical analyses were performed in the R software, version 3.4.2 [48].

## Results

### Benthic structure

**Fig 2:**
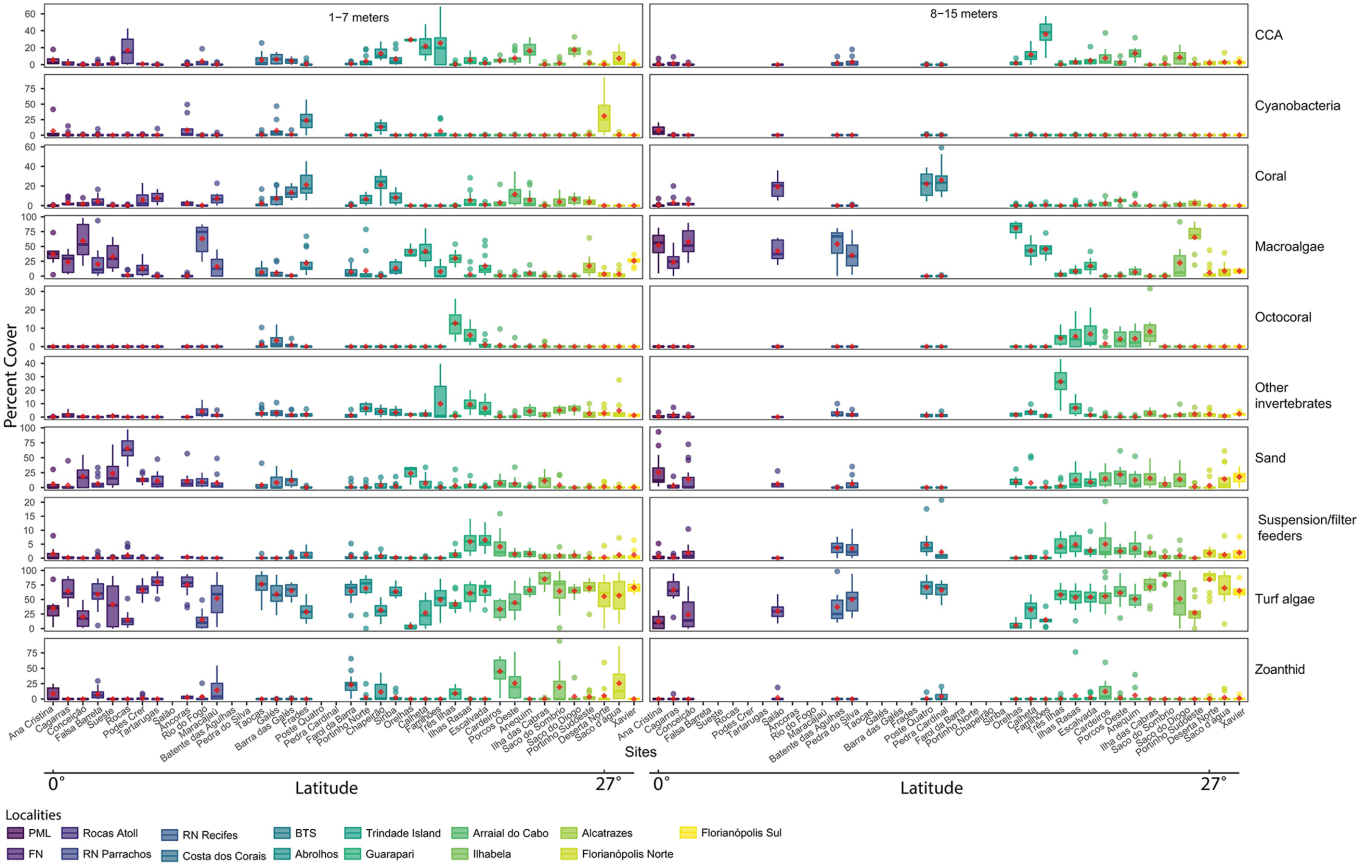
Percent cover of reef surface by sites at shallow (1–7 meters) and deep strata (8–15 meters). Bars represent the median, two hinges and two whiskers. Dots are outliers. Red dots represent the means. Sites are orientated from 0°latitude to 27°S latitude. Red dots represent the means. PML = Parcel do Manuel Luis, FN = Fernando de Noronha, BTS = Baia de Todos os Santos.

**Fig 3:**
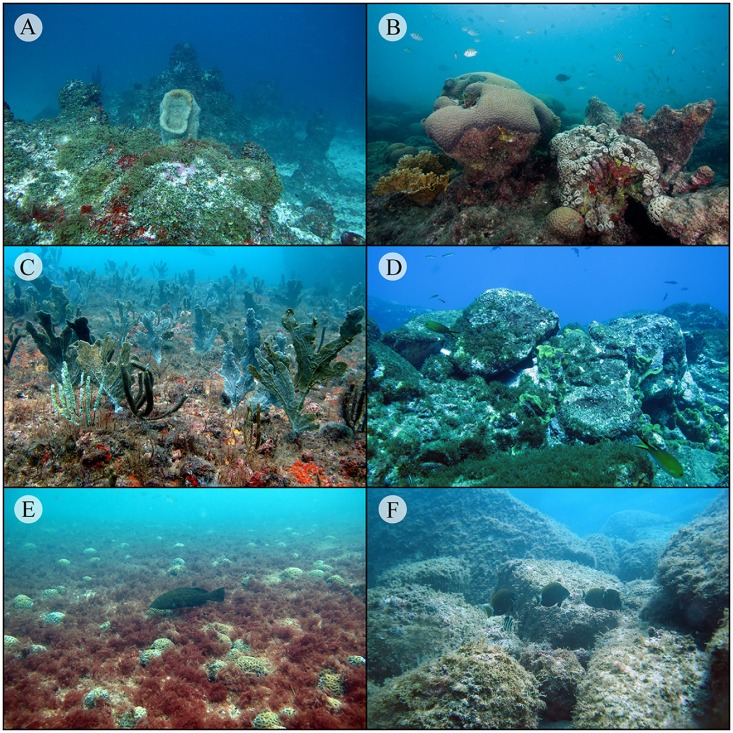
The general aspect of reef benthic communities in the Brazilian Province. **(A)** Parcel do Manuel Luis (PML), (**B)** Abrolhos, (**C)** Guarapari, (**D)** Trindade Island, (**E)** Alcatrazes and (**F)** Florianópolis Sul.

Benthic communities in the Brazilian Province were dominated by turf algae (mean cover = 52.9% ± 27.6 SD; Figs 1B and 2). The localities of Parcel do Manuel Luis (PML; 0°latitude), and Trindade Island (20°latitude) exhibited the lowest cover of turf algae (19.20% ± 19.82 SD and 23.35% ± 23.09 SD, respectively). On the other hand, the localities of Ilhabela (23°latitude), Florianópolis Sul (27°latitude) and Florianópolis Norte (27°latitude) had the highest turf algae cover (71.35% ± 25.18 SD, 68.34% ± 10.71 SD and 67.51% ± 27.70 SD, respectively; Figs 1B and 2). Frondose macroalgae were also abundant in the Brazilian Province (mean cover = 17.36% ± 24.04 SD; Figs 1B, 2 and 3), but was variable among localities (Trindade Island = 47.35% ± 27.18 SD (20°latitude) to Arraial do Cabo = 2.33% ± 4.05 SD (22°latitude)) and depth strata between sites (Fig 2). Localities distant from the coast showed the highest cover of frondose macroalgae (Trindade Island = 47.35% ± 27.18 SD (20°latitude) and PML = 47.34% ± 22.74 SD (0°latitude)), characterized by a high cover of *Halimeda* sp. at PML and *Caulerpa verticillata* at Trindade Island. Overall, 67.5% of the 40 sampled reefs were turf dominated (*i*.*e*. percent cover >50%), 12.5% of all reefs were dominated by frondose macroalgae, and 80% of the reefs were dominated by turf and frondose macroalgae (Fig 2).

Overall, cover of reef-building coral was low (mean cover = 4.38% ± 8.17 SD; Figs 1, 2 and 3) and dominated by massive species, mostly colonies in the genera *Siderastrea*, *Montastraea*, and *Mussismilia*. The highest coral percent cover occurred at Baia de Todos os Santos (BTS; 12°latitude), Abrolhos (17°latitude) and Costa dos Corais (9°latitude) (mean cover = 17.23% ± 15.07 SD, 12.05% ± 10.25 SD and 7.63% ± 7.46 SD, respectively; Fig 1B). Corals had higher percent cover at 1–7 meters (Fig 2). The percent cover of reef-builders (corals and CCA) was also low in most localities. The only localities that displayed percent cover of reef-builders greater than 5% were Costa dos Corais (9°latitude; 16.50% ± 6.67 SD), Abrolhos (17°latitude; 9.99% ± 9.26 SD), BTS (12°latitude; 8.90% ± 13.55 SD), Trindade Island (20°latitude; 8.84% ± 15.33 SD), and Arraial do Cabo (22°latitude; 6.96% ± 8.30 SD; Figs 1B and 2). At latitudes higher than 24°S (Alcatrazes), the percent cover of reef-builders was close to zero (mean cover = 1.55% ± 3.40 SD; Figs 1B and 2).

The percent cover of octocorals, suspension/filter feeders (mostly ascidians and sponges) and other invertebrates were low overall but increased at latitudes higher than 20°S (Guarapari; Figs 1B and 2). Octocorals and suspension/filter feeders were more abundant at 8–15 meters, while zoanthids were more abundant at 1–7 meters depth (Fig 2). Among all the localities, BTS (12°latitude), Abrolhos (17°latitude), Guarapari (20°latitude) and Arraial do Cabo (22°latitude) were characterized by a more diverse composition of benthic groups composition (Fig 1B). These localities all had approximately 50% of the substrate free of turf algae or frondose macroalgae. BTS and Abrolhos exhibited higher cover of reef-builders (mostly corals), zoanthids and cyanobacteria. In contrast, Guarapari and Arraial do Cabo showed a greater representation of zoanthids (dominated by *Palythoa caribaeorum*), reef-builders (mostly CCA), octocorals (*Phylogorgia dilatata*, *Plexaurella regia*, *Plexaurella grandiflora* and *Leptogorgia* sp.), suspension/filter feeders and other invertebrates (mostly crinoids).

**Fig 4:**
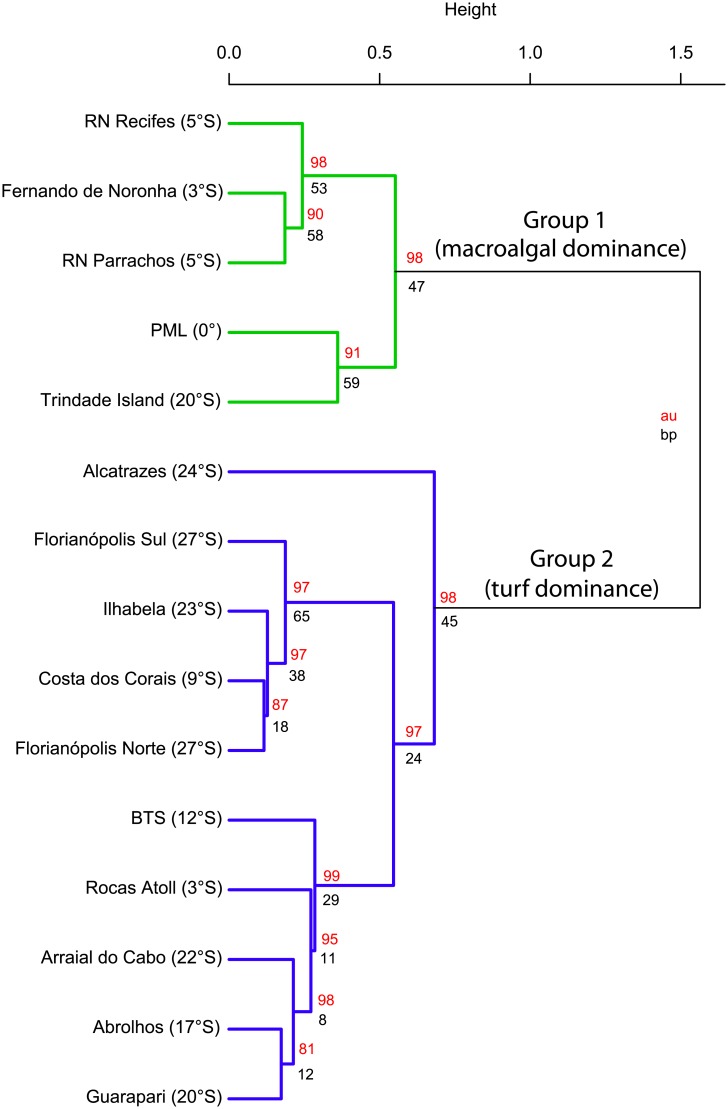
Cluster analysis (complete linkage method) of benthic cover at localities sampled. Approximated unbiased (red) and bootstrap probability (black) are the values of cophenetic correlation analysis. Green and blue show significant clades identified. PML = Parcel do Manuel Luis, BTS = Baia de Todos os Santos.

Regarding the geographic distribution of our sampled communities, the cluster analysis revealed two major cluster, grouped by frondose macroalgae abundance (Fig 4). The first group, with macroalgal dominance, was composed by the localities of RN Recifes (5°latitude), RN Parrachos (5°latitude), Fernando de Noronha (FN; 3°latitude), PML (0°latitude) and Trindade Island (20°latitude). The second group, dominated by turf algae, was formed by Alcatrazes (24°latitude), Florianópolis Sul (27°latitude), Ilhabela (23°latitude), Costa dos Corais (9°latitude), Florianópolis Norte (27°latitude), BTS (13°latitude), Rocas Atoll (3°latitude), Arraial do Cabo (22°latitude), Abrolhos (17°latitude), and Guarapari (20°latitude). Benthic community composition differed among localities but not between depth strata and reef type (Table 1; Figure A in S1 Fig). Benthic communities of the sites at Trindade Island, PML, Guarapari and FN were distinct from those at the other localities (NMDS; Figure B in S1 Fig).

**Table 1:** Results of PERMANOVA test of benthic communities, using arcsine-square root transformation and Bray-Curtis dissimilarities.

Source	df	F	p (perm)
Reef	1	1.6038	0.099
Locality	9	5.0275	0.001*
Depth	1	1.3302	0.198
Locality:Depth	8	0.8853	0.753
Residual	20		

Reef = reef type (biogenic and rocky reef), df = degree of freedom, F = F value, p = p value.

(*) indicate significant difference.

### Diversity patterns

A total of 103 taxa were recorded across the Brazilian Province. Both observed taxa and Chao estimator showed the same patterns (S2 Fig). We found low diversity at low latitudes (at latitude 5°S; S_obs_ = 12 and Chao = 13.43), while diversity peaked at mid-latitudes, around 20°S to 23°S (~3.5-fold higher than lower richness; Fig 1C and S2 Fig). Among the three oceanic islands, Rocas Atoll showed the highest diversity (S_obs_ = 34 and Chao = 41.92) and Trindade Island the lowest diversity (S_obs_ = 20 and Chao = 23.93) (Fig 1C and S2 Fig).

## Discussion

Our results provide the first broad-scale baseline of abundance and diversity patterns for shallow water benthic communities along the Brazilian coastline and oceanic islands. Reefs of the Brazilian Province have low reef-building coral cover and are dominated by algal turfs and macroalgae, even at biogenic reef systems, and among coastal and oceanic reef localities. High algae cover has been observed on reefs elsewhere, but not to the same extent as documented here for the Brazilian Province. For instance, macroalgae cover on Caribbean reefs is ~23.6% [55] and 1% of the reefs in the Indo-Pacific show macroalgae cover higher than 50% [56]. Turf algae were the most abundant benthic group on Curaçao reefs (percent cover ranging from 20.3–41%; [57]), in the Mediterranean (percent cover ranging from 50–70%; [58]), South Australia (percent cover of 39%; [59]), and at the remote reefs of the Line Islands (36% cover; [60]), but none of these studies recorded such a high cover of algal turfs as noted here.

Many reef studies have documented the decline of calcifying organisms (corals and CCA) and phase shifts to macroalgae and turf algae [29, 61–63]. Primary producers, such as macroalgae and turf algae, can benefit and become dominant when there is an increase in nutrients and sediment loads, and a reduction of herbivores [29, 57, 59, 64]. Turf algae, for example, can occupy space quickly by vegetative reproduction and become dominant under different disturbance and stress conditions [65]. In subtropical reefs of Arraial do Cabo, the aquarium trade collection was reported to cause the loss of 50% of coral cover, mainly fire corals [66], with turf algae being the most competitive group to occupy free space. Additionally, herbivorous fishes, like parrotfishes, were reported as overfished on southeastern Brazilian reefs [67–68]. This dominance of turf and macroalgae on Brazilian reefs may occur because (1) the physicochemical conditions of Brazilian waters and low coral cover may facilitate the high cover of turf algae and macroalgae, resulting in a different, potentially stable state for the community; (2) the effect of anthropogenic activities, such as reduction of herbivores and high sedimentation/nutrients inputs caused by urban development and coastal runoff may have resulted in a phase shift; or (3) a combination of physicochemical conditions and anthropogenic activities. Although studies have reported an increase of turf algae cover in the Caribbean (from 24.5% to 38%; [69]) and a moderate increase at the Abrolhos reef in Brazil [18], the lack of previous reports on Brazilian benthic community structure makes it difficult to determine if turf-dominated reefs in the Brazilian Province are a result of reef degradation or part of a different stable state.

Benthic community composition differed among localities, mostly due to algae composition, but did not follow a clear latitudinal pattern. Different benthic communities are usually associated with nutrient and light availability [70–71], differences in sea temperature and salinity [72] and effects of disturbances [73]. For example, PML (0° latitude), Trindade Island (20°S latitude) and FN (3°latitude) are distant from the coast and with clear waters, where light availability may influence the high cover of macroalgae. Arraial do Cabo, for instance, is influenced by upwelling events [74] which could affect benthic community structure [75]. Therefore, we suggest that a combination of local and context-dependent factors (*e*.*g*. water clarity, upwelling, urban development) may be driving the differences among the benthic communities of the studied localities.

We found low benthic diversity in the tropics, which differs from the general pattern of latitudinal gradient diversity. Although many marine taxa exhibit a global pattern of diversity peaking in the western Pacific and near the equator [6], many other studies have documented that latitudinal patterns in the Atlantic differ from this general diversity patterns for different groups of organisms [8, 25, 34]. The lower diversity in the tropics of the southwestern Atlantic has been attributed to a combination of extreme environmental conditions, such as high waves and wind exposure at the northeast part of Brazil, heterogeneous and narrow shelf width, and sedimentation and/or salinity effects from rivers [8, 38, 76]. Such factors can play an important role in the establishment and survival of reef organisms.

Instead of peaking near the equator, we found that the highest diversity (~3.5-fold greater than the most depauperate locality) in the Brazilian Province occurred at mid-latitudes, around 20°S to 23°S. This same pattern has been described for different taxonomic groups in the southwestern Atlantic, including fishes [77], algae, invertebrates and fish [25], gastropods [34], and *Symbiodinium* [78]. This mid-latitude region corresponds to a transitional zone between tropical and subtropical reefs influenced by the warm Brazil Current and the cold Brazilian Northern Current. This may allow organisms with tropical and subtropical affinities to coexist, resulting in higher diversity. Also, the heterogeneity of local habitats within this region (*e*.*g*. coralline communities, rocky reefs and rhodolith beds) has been suggested as a factor contributing to the greater diversity of reef organisms [34, 79–80].

Oceanic localities showed low diversity compared to coastal communities. Oceanic islands tend to display low species richness and high endemic rates as result of their isolation and relatively shallow water zones [81]. For example, Trindade Island showed a remarkably low richness despite its latitudinal position (20°S) and is considered one of the most species-poor oceanic islands in the world [12]. The large distance from the coast restricts immigration of species with limited dispersal abilities from the mainland. In addition, relatively narrow shallow zones, and strong oceanographic conditions (i.e. wave exposure, currents) may contribute to its low richness [82].

This is the first study to provide a standardized quantitative characterization of the shallow water benthic communities of the Brazilian Province. We demonstrated that algal turfs and macroalgae are the dominant groups across the Province. The absence of any previous quantitative baselines on this scale limits our ability to determine if this is a natural stable state of Brazilian marine communities, or a result of anthropogenic effects, or a combination of both. Future experimental and observational studies are needed to properly address this issue. The baseline information on benthic community composition presented here can be used for macroecological studies and to evaluate impacts in Brazilian marine habitats, such as the impact of a mining dam collapse in Doce river [83] that has affected sediment and water quality at Guarapari and Abrolhos regions [84–85]. Also, our results on benthic diversity patterns can contribute to the discussion on future environmental planning and management targets. The coastal region around 20°S to 23°S holds the highest diversity of fish [86] and we show that this region also has the highest benthic diversity. However, this region contains few marine protected areas (MPA), resulting in a general mismatch among MPA locations and reef biodiversity. Thus, combining the results for reef benthic communities presented here with reef fish diversity [86], we can improve the understanding of spatial patterns in marine biodiversity, an essential first step for establishing MPAs.

## Supporting information

S1 Table Summary of field effort at the sites along the Brazilian Province. FN = Fernando de Noronha, PML = Parcel do Manuel Luis, BTS = Baia de Todos os Santos.
(PDF)

S2 Table Classification of taxa and benthic group related. (PDF)

S1 Fig nMDS ordination with the benthic community composition. **(A) sites only with both depth strata; (B) all sites surveyed**. Black squares are the organisms. FN = Fernando de Noronha, PML = Parcel do Manuel Luis and BTS = Baia de Todos os Santos.
(EPS)

S2 Fig Species accumulation curves using observed taxa (S_obs_) and Chao estimator. (EPS)
