# Supplementary material for: Large-scale patterns of benthic marine communities in the Brazilian Province
Source: PLoS One. 2018 Jun 8;13(6):e0198452. doi: 10.1371/journal.pone.0198452 (PMC5993233; doi:10.1371/journal.pone.0198452)
Supplement: S1 Table — FN = Fernando de Noronha, PML = Parcel do Manuel Luis, BTS = Baia de Todos os Santos. (PDF) [file pone.0198452.s001.pdf]

Locality	Site	Lat	Lon	Reef type	Month	Year	Depth (m)	N (2m^2 area)	N (photoquadrats)
Rocas Atoll	Âncoras	-3.87517	-33.8036	Biogenic	January	2012	1-7	16	80
Rocas Atoll	Falsa Barreta	-3.86035	-33.8188	Biogenic	January	2012	1-7	19	95
Rocas Atoll	Rocas	-3.86895	-33.7919	Biogenic	January	2012	1-7	20	100
Rocas Atoll	Podes Crer	-3.87296	-33.8123	Biogenic	January	2012	1-7	17	85
Rocas Atoll	Salão	-3.87473	-33.8094	Biogenic	January	2012	8-15	9	45
Rocas Atoll	Tartarugas	-3.87299	-33.8094	Biogenic	January	2012	1-7	18	90
FN	Cagarras	-3.81455	-32.3902	Rocky	October	2011	1-7	13	65
							8-15	15	75
FN	Conceição	-3.83904	-32.415	Rocky	October	2011	1-7	14	70
							8-15	15	75
FN	Sueste	-3.86684	-32.4228	Rocky	October	2011	1-7	14	70
Trindade Island	Calheta	-20.5075	-29.3103	Rocky	July	2012	1-7	11	55
							8-15	13	65
Trindade Island	Farrilhões	-20.5226	-29.4979	Rocky	July	2012	1-7	9	45
							8-15	8	40
Trindade Island	Orelhas	-20.4925	-29.3431	Rocky	July	2012	1-7	4	20
							8-15	14	70
PML	Ana Cristina	-0.86987	-44.2643	Biogenic	April	2013	1-7	7	35
							8-15	14	70
RN Parrachos	Maracajaú	-5.39411	-35.259	Biogenic	October	2011	1-7	14	70
RN Parrachos	Rio do Fogo	-5.26212	-35.3634	Biogenic	October	2011	1-7	8	40
RN Recifes	Batente das Agulhas	-5.56435	-35.0725	Biogenic	March	2013	8-15	8	40
RN Recifes	Pedra do Silva	-5.56447	-35.0901	Biogenic	March	2013	8-15	14	70
Costa dos Corais	Barra das Galés	-9.03269	-35.1927	Biogenic	March	2012	1-7	15	75
Costa dos Corais	Galés	-9.02426	-35.1915	Biogenic	March	2012	1-7	15	75
Costa dos Corais	Taocas	-8.9985	-35.1806	Biogenic	March	2012	1-7	15	75
BTS	Farol da Barra	-13.0083	-38.5308	Biogenic	March	2012	1-7	15	75
BTS	Frades	-12.8092	-38.6264	Biogenic	March	2012	1-7	15	75

Locality	Site	Lat	Lon	Reef type	Month	Year	Depth (m)	N (2m^2 area)	N (photoquadrats)
BTS	Pedra Cardinal	-12.8371	-38.5491	Biogenic	March	2012	8-15	14	70
BTS	Poste Quatro	-12.8147	-38.5717	Biogenic	March	2012	8-15	15	75
Abrolhos	Chapeirão	-17.9628	-38.6625	Biogenic	March	2010	1-7	15	75
Abrolhos	Portinho Norte	-17.9638	-38.6936	Biogenic	March	2010	1-7	15	75
Abrolhos	Siriba	-17.9706	-38.7158	Biogenic	March	2010	1-7	15	75
Guarapari	Escalvada	-20.6996	-40.4076	Rocky	February	2014	1-7	15	75
							8-15	15	75
Guarapari	Ilhas Rasas	-20.6766	-40.3662	Rocky	February	2014	1-7	15	75
							8-15	15	75
Guarapari	Três Ilhas	-20.6123	-40.3788	Rocky	February	2014	1-7	15	75
							8-15	15	75
Arraial do Cabo	Anequim	-22.9805	-41.9845	Rocky	March	2011	1-7	14	70
							8-15	10	50
Arraial do Cabo	Cardeiros	-22.9651	-42.0017	Rocky	March	2011	1-7	16	80
							8-15	15	75
Arraial do Cabo	Porcos Oeste	-22.9657	-41.9937	Rocky	March	2011	1-7	13	65
							8-15	8	40
Ilhabela	Ilhas das Cabras	-23.8303	-45.3939	Rocky	March	2013	1-7	13	65
							8-15	14	70
Ilhabela	Saco do Diogo	-23.9354	-45.2836	Rocky	March	2013	1-7	9	45
							8-15	13	65
Ilhabela	Saco do Sombrio	-23.8933	-45.244	Rocky	March	2013	1-7	11	55
							8-15	11	55
Alcatrazes	Portinho Sudoeste	-24.1052	-45.7025	Rocky	March	2013	1-7	12	60
							8-15	10	50
Florianópolis Norte	Saco d'água	-27.277	-48.3685	Rocky	April	2011	1-7	10	50
							8-15	14	70
Florianópolis Norte	Deserta Norte	-27.2645	-48.3317	Rocky	April	2011	1-7	15	75

Locality	Site	Lat	Lon	Reef type	Month	Year	Depth (m)	N (2m^2 area)	N (photoquadrats)
							8-15	15	75
Florianópolis Sul	Xavier	-27.6035	-48.3876	Rocky	April	2011	1-7	8	40
							8-15	6	30
**Total**								**764**	**3820**
