# Supplementary material for: Large-scale patterns of benthic marine communities in the Brazilian Province
Source: PLoS One. 2018 Jun 8;13(6):e0198452. doi: 10.1371/journal.pone.0198452 (PMC5993233; doi:10.1371/journal.pone.0198452)
Supplement: S2 Table — (PDF) [file pone.0198452.s002.pdf]

sisbiota_code	Taxa	Kingdom	Phylum	Class	Order	Family	Genus	Benthic Group
Agaricia.fragilis	*Agaricia fragilis*	Animalia	Cnidaria	Anthozoa	Scleractinia	Agariciidae	*Agaricia*	Coral
Agaricia.humilis	*Agaricia humilis*	Animalia	Cnidaria	Anthozoa	Scleractinia	Agariciidae	*Agaricia*	Coral
Agaricia.sp	*Agaricia* spp	Animalia	Cnidaria	Anthozoa	Scleractinia	Agariciidae	*Agaricia*	Coral
Anemona	*Bunodosoma caissarum*	Animalia	Cnidaria	Anthozoa	Actiniaria	Actiniidae	*Bunodosoma*	Other invertebrates
Arborescent.sponge	Demospongiae	Animalia	Porifera	Demospongiae				Suspension/filter feeders
Ascidea.colonial	Phlebobranchia	Animalia	Chordata	Ascidiacea	Phlebobranchia			Suspension/filter feeders
Astrangia.Phyllangia	Rhizangiidae	Animalia	Cnidaria	Anthozoa	Scleractinia	Rhizangiidae		Coral
Botrylloides.nigrum	*Botrylloides nigrum*	Animalia	Chordata	Ascidiacea	Stolidobranchia	Styelidae	*Botrylloides*	Suspension/filter feeders
Bryopsis.pennata	*Bryopsis pennata*	Plantae	Chlorophyta	Ulvophyceae	Bryopsidales	Bryopsidaceae	*Bryopsis*	Macroalgae
calcareous.articulate.algae	Corallinaceae	Plantae	Rhodophyta	Rhodophyceae	Corallinales	Corallinaceae		Macroalgae
calcareous.turf	Coralline turf	Plantae	Rhodophyta	Rhodophyceae	Corallinales	Corallinaceae		Turf
Carijoa.riisei	*Carijoa riisei*	Animalia	Cnidaria	Anthozoa	Alcyonacea	Clavulariidae	*Carijoa*	Octocoral
Caulerpa.racemosa	*Caulerpa racemosa*	Plantae	Chlorophyta	Ulvophyceae	Bryopsidales	Caulerpaceae	*Caulerpa*	Macroalgae
Caulerpa.sp	*Caulerpa* spp.	Plantae	Chlorophyta	Ulvophyceae	Bryopsidales	Caulerpaceae	*Caulerpa*	Macroalgae
Caulerpa.verticillata	*Caulerpa verticillata*	Plantae	Chlorophyta	Ulvophyceae	Bryopsidales	Caulerpaceae	*Caulerpa*	Macroalgae
Chaetomorpha.sp	*Chaetomorpha*	Plantae	Chlorophyta	Ulvophyceae	Cladophorales	Cladophoraceae	*Chaetomorpha*	Macroalgae
Champia.parvula	*Champia parvula*	Plantae	Chlorophyta	Florideophyceae	Rhodymeniales	Champiaceae	*Champia*	Macroalgae
Cianobacterias	Cyanobacteria	Bacteria	Cyanobacteria					Cyanobacteria
Cirripedia	Hexanauplia	Animalia	Arthropoda	Hexanauplia				Other invertebrates
Codium.intertextum	*Codium intertextum*	Plantae	Chlorophyta	Ulvophyceae	Bryopsidales	Codiaceae	*Codium*	Macroalgae
Codium.spp	*Codium* spp.	Plantae	Chlorophyta	Ulvophyceae	Bryopsidales	Codiaceae	*Codium*	Macroalgae
Colpomenia.sinuosa	*Colpomenia sinuosa*	Chromista	Ochrophyta	Phaeophyceae	Scytosiphonales	Scytosiphonaceae	*Colpomenia*	Macroalgae
corticated.algae	Gigartinacea	Plantae	Rhodophyta	Florideophyceae	Gigartinales	Gigartinacea		Macroalgae
Crinóide	*Tropiometra* sp.	Animalia	Echinodermata	Crinoidea	Comatulida	Tropiometridae	*Tropiometra*	Other invertebrates
crostose.coralline.algae	Corallinales	Plantae	Rhodophyta	Florideophyceae	Corallinales			CCA
Dictyopteris	*Dictyopteris* spp.	Chromista	Ochrophyta	Phaeophyceae	Dictyotales	Dictyotaceae	*Dictyopteris*	Macroalgae
Dictyopteris.plagiogramma	*Dictyopteris plagiogramma*	Chromista	Ochrophyta	Phaeophyceae	Dictyotales	Dictyotaceae	*Dictyopteris*	Macroalgae
Dictyota.sp	*Dictyota* spp.	Chromista	Ochrophyta	Phaeophyceae	Dictyotales	Dictyotaceae	*Dictyota*	Macroalgae

sisbiota_code	Taxa	Kingdom	Phylum	Class	Order	Family	Genus	Benthic Group
Didemnum	*Didemnum* sp.	Animalia	Chordata	Ascidiacea	Aplousobranchia	Didemnidae	*Didemnum*	Suspension/filter feeders
Didemnum.perlucidum	*Didemnum perlucidum*	Animalia	Chordata	Ascidiacea	Aplousobranchia	Didemnidae	*Didemnum*	Suspension/filter feeders
Digenea.sp.	*Digenea simplex*	Plantae	Rhodophyta	Florideophyceae	Ceramiales	Rhodomelaceae	*Digenea*	Macroalgae
Estrela	*Echinaster*	Animalia	Echinodermata	Asteroidea	Spinulosida	Echinasteridae	*Echinaster*	Other invertebrates
Favia.gravida	*Favia gravida*	Animalia	Cnidaria	Anthozoa	Scleractinia	Mussidae	*Favia*	Coral
foliaceous.algae	Dictyotaceae	Chromista	Ochrophyta	Phaeophyceae	Dictyotales	Dictyotaceae		Macroalgae
Galaxaura.sp	*Galaxaura* spp.	Plantae	Rhodophyta	Florideophyceae	Nemaliales	Galaxauraceae	*Galaxaura*	Macroalgae
Gelidiella.acerosa	*Gelidiella acerosa*	Plantae	Rhodophyta	Florideophyceae	Gelidiales	Gelidiellaceae	*Gelidiella*	Macroalgae
Gelidiopsis	*Gelidiopsis* spp.	Plantae	Rhodophyta	Florideophyceae	Rhodymeniales	Lomentariaceae	*Gelidiopsis*	Macroalgae
Gelidium.floridanum	*Gelidium floridanum*	Plantae	Rhodophyta	Florideophyceae	Gelidiales	Gelidiaceae	*Gelidium*	Macroalgae
Globular.sponge	Demospongiae	Animalia	Porifera	Demospongiae				Suspension/filter feeders
green.filamentous.algae	Ulvophyceae	Plantae	Chlorophyta	Ulvophyceae				Macroalgae
Halimeda	*Halimeda* spp.	Plantae	Chlorophyta	Ulvophyceae	Bryopsidales	Halimedaceae	*Halimeda*	Macroalgae
Heterogorgia	*Heterogorgia* spp.	Animalia	Cnidaria	Anthozoa	Alcyonacea	Plexauridae	*Heterogorgia*	Octocoral
Hypnea.musciformis	*Hypnea musciformis*	Plantae	Rhodophyta	Florideophyceae	Gigartinales	Cystocloniaceae	*Hypnea*	Macroalgae
Idiellana.pristis	*Idiellana pristis*	Animalia	Cnidaria	Hydrozoa	Leptothecata	Sertulariidae	*Idiellana*	Other invertebrates
Incrusting.sponge	Demospongiae	Animalia	Porifera	Demospongiae				Suspension/filter feeders
Jania.Amphiroa	Corallinaceae	Plantae	Rhodophyta	Florideophyceae	Corallinales	Corallinaceae		Macroalgae
Laurencia.sp	*Laurencia* spp.	Plantae	Rhodophyta	Florideophyceae	Ceramiales	Rhodomelaceae	*Laurencia*	Macroalgae
leathery.algae	Dictyotaceae	Chromista	Ochrophyta	Phaeophyceae	Dictyotales	Dictyotaceae		Macroalgae
Leptogorgia.sp	*Leptogorgia* spp.	Animalia	Cnidaria	Anthozoa	Alcyonacea	Gorgoniidae	*Leptogorgia*	Octocoral
Lobophora.variegata	*Lobophora variegata*	Chromista	Ochrophyta	Phaeophyceae	Dictyotales	Dictyotaceae	*Lobophora*	Macroalgae
Macrorhynchia.philippina	*Macrorhynchia philippina*	Animalia	Cnidaria	Hydrozoa	Leptothecata	Aglaopheniidae	*Macrorhynchia*	Other invertebrates
Madracis.decactis	*Madracis decactis*	Animalia	Cnidaria	Anthozoa	Scleractinia	Astrocoeniidae	*Madracis*	Coral
Massive.sponge	Demospongiae	Animalia	Porifera	Demospongiae				Suspension/filter feeders
Meandrina.brasiliensis	*Meandrina brasiliensis*	Animalia	Cnidaria	Anthozoa	Scleractinia	Meandrinidae	*Meandrina*	Coral
Millepora.alcicornis	*Millepora alcicornis*	Animalia	Cnidaria	Hydrozoa	Anthoathecata	Milleporidae	*Millepora*	Coral
Millepora.incrusting	*Millepora* sp.	Animalia	Cnidaria	Hydrozoa	Anthoathecata	Milleporidae	*Millepora*	Coral

sisbiota_code	Taxa	Kingdom	Phylum	Class	Order	Family	Genus	Benthic Group
Millepora.nitida	*Millepora nitida*	Animalia	Cnidaria	Hydrozoa	Anthoathecata	Milleporidae	*Millepora*	Coral
Millepora.sp	*Millepora* spp.	Animalia	Cnidaria	Hydrozoa	Anthoathecata	Milleporidae	*Millepora*	Coral
Montastraea.cavernosa	*Montastraea cavernosa*	Animalia	Cnidaria	Anthozoa	Scleractinia	Montastraeidae	*Montastraea*	Coral
Muricea.flamma	*Muricea flamma*	Animalia	Cnidaria	Anthozoa	Alcyonacea	Plexauridae	*Muricea*	Octocoral
Muriceopsis.sulphurea	*Muriceopsis sulphurea*	Animalia	Cnidaria	Anthozoa	Alcyonacea	Plexauridae	*Muriceopsis*	Octocoral
Mussismilia.braziliensis	*Mussismilia braziliensis*	Animalia	Cnidaria	Anthozoa	Scleractinia	Mussidae	*Mussismilia*	Coral
Mussismilia.harttii	*Mussismilia harttii*	Animalia	Cnidaria	Anthozoa	Scleractinia	Mussidae	*Mussismilia*	Coral
Mussismilia.hispida	*Mussismilia hispida*	Animalia	Cnidaria	Anthozoa	Scleractinia	Mussidae	*Mussismilia*	Coral
Mussismilia.leptophylla	*Favia leptophylla*	Animalia	Cnidaria	Anthozoa	Scleractinia	Mussidae	*Favia*	Coral
Mussismilia.spp	*Mussismilia* spp.	Animalia	Cnidaria	Anthozoa	Scleractinia	Mussidae	*Mussismilia*	Coral
Octhodes.secundiramea	*Ochtodes secundiramea*	Plantae	Rhodophyta	Florideophyceae	Gigartinales	Rhizophyllidaceae	*Ochtodes*	Macroalgae
Octocoral.another	Alcyonacea	Animalia	Cnidaria	Anthozoa	Alcyonacea			Octocoral
Ophiothela.mirabilis	*Ophiothela mirabilis*	Animalia	Echinodermata	Ophiuroidea	Ophiurida	Ophiotrichidae	*Ophiothela*	Other invertebrates
ouriço1	*Echinometra lucunter*	Animalia	Echinodermata	Echinoidea	Camarodonta	Echinometridae	*Echinometra*	Other invertebrates
ouriço2	*Eucidaris tribuloides*	Animalia	Echinodermata	Echinoidea	Cidaroida	Cidaridae	*Eucidaris*	Other invertebrates
Outra.ascidia	Phlebobranchia	Animalia	Chordata	Ascidiacea	Phlebobranchia			Suspension/filter feeders
Outro.anthozoa	Anthozoa	Animalia	Cnidaria	Anthozoa				Other invertebrates
Outro.crustaceo	Malacostraca	Animalia	Arthropoda	Malacostraca				Other invertebrates
Outro.echinoderma	Asteroidea	Animalia	Echinodermata	Asteroidea				Other invertebrates
Outro.hydrozoa	Hydrozoa	Animalia	Cnidaria	Hydrozoa				Other invertebrates
Padina.sp	*Padina*	Chromista	Ochrophyta	Phaeophyceae	Dictyotales	Dictyotaceae	*Padina*	Macroalgae
Palythoa.caribaeorum	*Palythoa caribaeorum*	Animalia	Cnidaria	Anthozoa	Zoantharia	Sphenopidae	*Palythoa*	Zoanthid
Palythoa.variabilis	*Palythoa variabilis*	Animalia	Cnidaria	Anthozoa	Zoantharia	Sphenopidae	*Palythoa*	Zoanthid
Papilate.sponge	Demospongiae	Animalia	Porifera	Demospongiae				Suspension/filter feeders
Parazoanthus.cf.axinellae	*Parazoanthus axinellae*	Animalia	Cnidaria	Anthozoa	Zoantharia	Parazoanthidae	*Parazoanthus*	Zoanthid
Peyssonnelia	*Peyssonnelia* sp.	Plantae	Rhodophyta	Florideophyceae	Peyssonneliales	Peyssonneliaceae	*Peyssonnelia*	Macroalgae
Phallusia.nigra	*Phallusia nigra*	Animalia	Chordata	Ascidiacea	Phlebobranchia	Ascidiidae	*Phallusia*	Suspension/filter feeders
Phyllogorgia.dilatata	*Phyllogorgia dilatata*	Animalia	Cnidaria	Anthozoa	Alcyonacea	Gorgoniidae	*Phyllogorgia*	Octocoral
Plexaurella.grandiflora	*Plexaurella grandiflora*	Animalia	Cnidaria	Anthozoa	Alcyonacea	Plexauridae	*Plexaurella*	Octocoral

sisbiota_code	Taxa	Kingdom	Phylum	Class	Order	Family	Genus	Benthic Group
Plexaurella.regia	*Plexaurella regia*	Animalia	Cnidaria	Anthozoa	Alcyonacea	Plexauridae	*Plexaurella*	Octocoral
Poliqueta	Polychaeta	Animalia	Annelida	Polychaeta				Other invertebrates
Porites.astreoides	*Porites astreoides*	Animalia	Cnidaria	Anthozoa	Scleractinia	Poritidae	*Porites*	Coral
Porites.branneri	*Porites branneri*	Animalia	Cnidaria	Anthozoa	Scleractinia	Poritidae	*Porites*	Coral
Porites.sp	*Porites* spp.	Animalia	Cnidaria	Anthozoa	Scleractinia	Poritidae	*Porites*	Coral
Protopalythoa	*Protopalythoa* spp.	Animalia	Cnidaria	Anthozoa	Zoantharia	Sphenopidae	*Protopalythoa*	Zoanthid
Sargassum.sp	*Sargassum* spp.	Chromista	Ochrophyta	Phaeophyceae	Fucales	Sargassaceae	*Sargassum*	Macroalgae
Schizoporella.sp	*Schizoporella* sp.	Animalia	Bryozoa	Gymnolaemata	Cheilostomatida	Schizoporellidae	*Schizoporella*	Other invertebrates
Siderastrea.spp	*Siderastrea* spp.	Animalia	Cnidaria	Anthozoa	Scleractinia	Siderastreidae	*Siderastrea*	Coral
Stypopodium	*Stypopodium* spp.	Chromista	Ochrophyta	Phaeophyceae	Dictyotales	Dictyotaceae	*Stypopodium*	Macroalgae
Tricleocarpa.cylindrica	*Tricleocarpa cylindrica*	Plantae	Rhodophyta	Florideophyceae	Nemaliales	Galaxauraceae	*Tricleocarpa*	Macroalgae
Trididemnum	*Trididemnum* sp.	Animalia	Chordata	Ascidiacea	Aplousobranchia	Didemnidae	*Trididemnum*	Suspension/filter feeders
Tubular.sponge	Demospongiae	Animalia	Porifera	Demospongiae				Suspension/filter feeders
Udotea	*Udotea* sp.	Plantae	Chlorophyta	Ulvophyceae	Bryopsidales	Udoteaceae	*Udotea*	Macroalgae
Ulvophyceae	Ulvophyceae	Plantae	Chlorophyta	Ulvophyceae				Macroalgae
Ventricaria.ventricosa	*Ventricaria ventricosa*	Plantae	Chlorophyta	Ulvophyceae	Siphonocladales	Valoniaceae	*Ventricaria*	Macroalgae
Wrangelia	*Wrangelia* sp.	Plantae	Rhodophyta	Florideophyceae	Ceramiales	Wrangeliaceae	*Wrangelia*	Macroalgae
Zoanthus.sociatus	*Zoanthus sociatus*	Animalia	Cnidaria	Anthozoa	Zoantharia	Zoanthidae	*Zoanthus*	Zoanthid
